# Right Bundle Branch Block and Rehospitalization in Patients With Heart Failure

**DOI:** 10.7759/cureus.66238

**Published:** 2024-08-05

**Authors:** Takahide Sano, Shunsuke Kiuchi, Shinji Hisatake, Takanori Ikeda

**Affiliations:** 1 Division of Cardiovascular Medicine, Department of Internal Medicine, Toho University Graduate School of Medicine, Tokyo, JPN

**Keywords:** prognosis, rehospitalization, right bundle branch block, electrocardiography, heart failure

## Abstract

Objective: The most important aspect of managing heart failure (HF) is preventing rehospitalization. Bundle branch block (BBB), particularly left BBB (LBBB), has been a known risk factor for worsening prognosis, whereas no such consideration has been made for right BBB (RBBB). However, recent research has shown that RBBB was associated with increased mortality. This study evaluated the effects of RBBB on prognosis, especially rehospitalization, in patients with HF.

Materials: This study included 698 patients admitted for HF. Those who died in the hospital (n = 31) and dropped out during observation (n = 143) were excluded. After one year of observation, the patients were divided into a control group (n = 361) and a major adverse cardiovascular event (MACE) group (n = 163). After further excluding according to electrocardiography findings, patients were categorized as having no BBB (n = 307), pure RBBB (n = 37), and LBBB (n = 56), and then the characteristics, clinical data, and prognosis of the remaining patients were evaluated.

Results: Patients were compared to no BBB, pure RBBB, and LBBB was associated with a risk for HF rehospitalization (p = 0.007). Furthermore, pure RBBB was independently associated with HF rehospitalization even after adjusting for confounders (hazard ratio: 2.40 (95% confidence interval: 1.26-4.58; p = 0.008).

Conclusion: Pure RBBB was independently associated with HF rehospitalization, highlighting the need for vigilance against the risk of HF rehospitalization among those with pure RBBB.

## Introduction

Heart failure (HF) is one of the most common chronic diseases in elderly individuals, a phenomenon called the HF pandemic [1]. The most important aspect of the management of HF is preventing rehospitalization. Understanding the risk factors for rehospitalization due to HF can lead to an improved HF prognosis [2]. A wide QRS duration or left bundle branch block (LBBB) on electrocardiography (ECG) has generally been known as a risk factor for mortality and rehospitalization due to HF [3]; however, there is no conclusion that right bundle branch block (RBBB) is same as LBBB [3] [4]. RBBB is induced by blockage of the Purkinje fibers in the right bundle branch caused by structural disorders (e.g., ischemic heart disease, valvular disease) [5]. RBBB is often observed in healthy individuals who remain almost asymptomatic and do not require further evaluation or treatments [5,6]. Approximately 1.5% of individuals in Japan have been diagnosed with RBBB on ECG during health examinations, a figure similar to that reported in Europe [5,7]. However, recent research indicates that the presence of RBBB was associated with increased mortality [6,8] in patients with cardiovascular disease (CVD). The current study aimed to evaluate the impact of RBBB on clinical outcomes, especially rehospitalization, in patients with HF throughout Japan.

## Materials and methods

Study design and population

**Figure 1:**
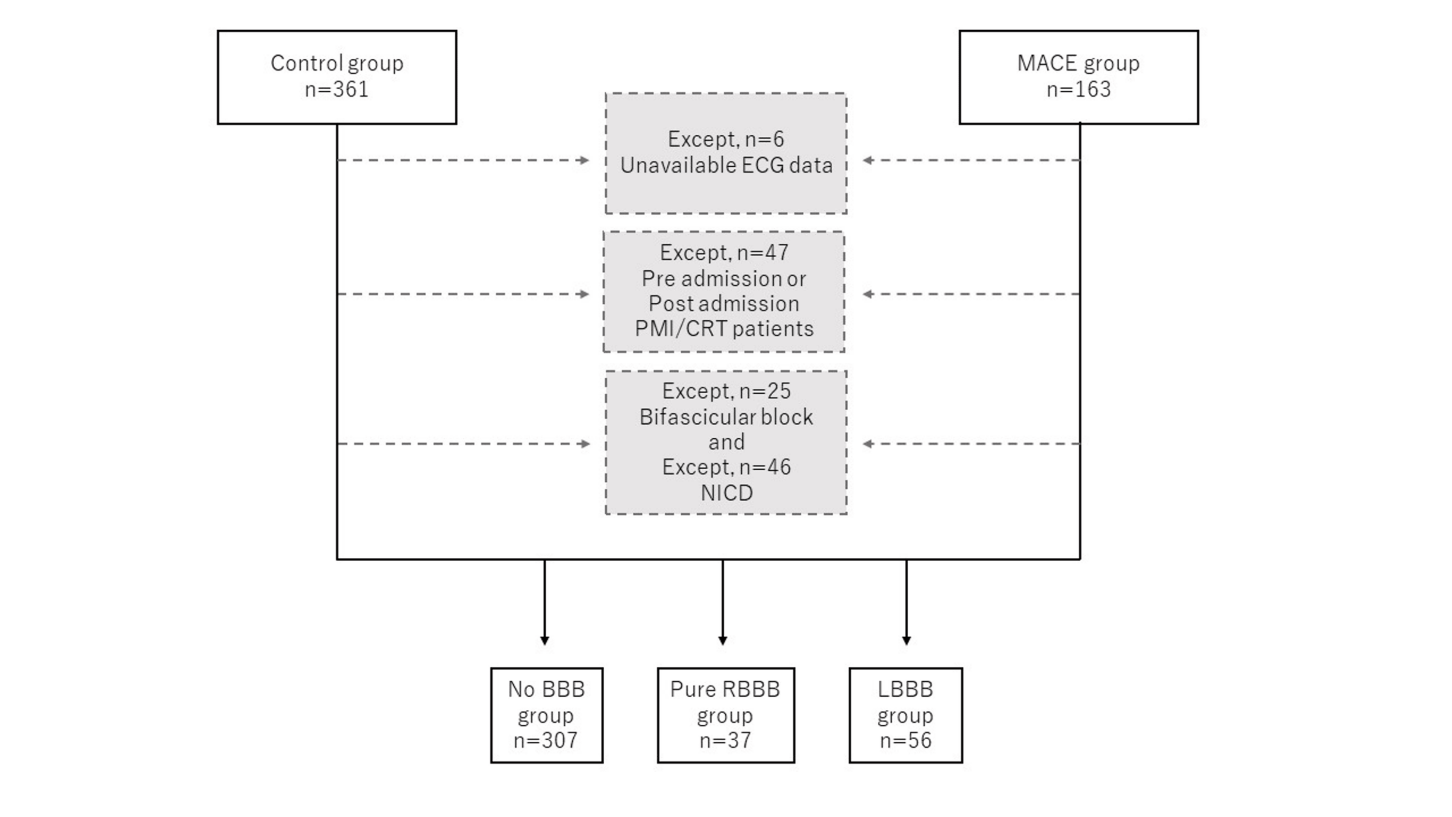
Study population Abbreviations: MACE = major adverse cardiovascular events; ECG = electrocardiogram; PMI = pacemaker implantation; CRT = cardiac resynchronization therapy; NICD = nonspecific intraventricular conduction delay; BBB = bundle branch block; RBBB = right bundle branch block; LBBB = left bundle branch block

This retrospective study included consecutive patients with HF admitted at Toho University Omori Medical Center, Ota Ward, Japan, from April 01, 2014, to March 31, 2018. Those in the acute phase of illness were classified using the diagnosis procedure combination (DPC) method on admission. This approach is used to standardize Japanese medical care and the reimbursement system [9]. DPC data were used to extract patients with HF, ultimately identifying 698 patients. After reviewing their medical records, patients who died in the hospital (n = 31) were excluded. The American Heart Association and/or European Society of Cardiology guidelines were used to diagnose HF [10,11]. The remaining patients were then categorized into a major adverse cardiovascular event (MACE) group (n = 163, 24.4%), which contains all causes of death, HF rehospitalization, acute coronary syndrome, and cerebral apoplexy, comprising those who developed MACE within one year and a control group (n = 361, 54.1%) comprising those who did not develop MACE over one year. Further subject selection was performed according to ECG results (Figure 1). The primary endpoint was MACE within one year of discharge, whereas the secondary endpoint was rehospitalization due to HF within one year of discharge. The subjects for this study were disclosed and provided the opportunity to decline enrollment in the study by opt-out. Because data were evaluated retrospectively and pseudonymously and were solely obtained for treatment purposes, the requirement of informed consent was exempted by the Ethics Committee of the Toho University Omori Medical Center. The study protocol, including the use of an opt-out method, was approved by the Toho University Omori Medical Center Ethics Committee (approval number: M23201). This study was conducted in accordance with the Declaration of Helsinki.

Data collection

Electronic medical records were used to determine the patient’s clinical characteristics, including symptoms, demographics, medical history, medications, baseline comorbidities, physical findings, laboratory test results, electrocardiography findings, cardiac echocardiography findings, treatment information, and outcomes.

Electrocardiography

A standard 12-lead ECG was performed using an ECG-2450 or ECG-2550 electrocardiograph (Nihon Koden, Inc., 1-32-4 Nishiochiai Shinjuku ward, Japan) at a paper speed of 25 mm/s upon admission or after hospitalization. When performed more than once, the procedure closest to the date of admission was used. ECG findings were accessed through the physiology laboratory database, and the PQ, QRS, QT, and QTc durations were automatically analyzed. The bundle branch block (BBB) was assessed after excluding the pacemaker rhythm. RBBB was defined as a QRS duration of ≥100 ms: rsR′, rsR′, rSR′, or qR patterns in leads V1 or V2; an S wave of greater duration than the R wave or >40 ms in leads I and V6; and normal R peak time in leads V5 and V6 but >50 ms in lead V1. Similarly, LBBB was defined as a QRS duration ≥100 ms: a broad notched or slurred R wave in leads I, aVL, V5, and V6; R peak time >60 ms in leads V4, V5, and V6; and the absence of a Q wave in leads I, V5, and V6. Nonspecific intraventricular conduction delay (NICD) was defined as a QRS duration ≥100 ms and the absence of morphology criteria for RBBB or LBBB. Bifascicular block was defined as a combination of RBBB with left anterior fascicular block or left posterior fascicular block assessed using axis deviation. Therefore, patients with RBBB were divided into those with pure RBBB and bifascicular block. Given that data obtained from the medical records were only used to evaluate BBB, ECG records for several patients were not captured from the physiology laboratory database. The patients were then classified into those with no BBB, LBBB, pure RBBB, bifascicular block, and NICD based on their ECG findings. After excluding those with unavailable ECG data (six patients), implanted pacemaker or cardiac resynchronization therapy (CRT, 47 patients), bifascicular block (25 patients), and NICD (46 patients), the remaining patients were categorized into the no BBB (307 patients), LBBB (56 patients), and pure RBBB (37 patients) groups and evaluated (Figure 1). All patients with complete atrioventricular block underwent pacemaker implantation while hospitalized and were classified as the implanted pacemaker group in the current study.

Statistical analysis

Categorical variables were presented as frequency and percentage (%) and evaluated using Fisher’s exact test, whereas continuous variables were presented as mean (±standard deviation) or median with interquartile range (quartiles 1-3) and evaluated using the Kruskal-Wallis test. Univariate and multivariate logistic regression analysis models were created to estimate the impact of RBBB on outcomes. The multivariate logistic regression model was adjusted using classical HF risk factors (body mass index (BMI), hemoglobin level, estimated glomerular filtration rate (eGFR) level, albumin level) [12-15]. The Kaplan-Meier method and Cox proportional hazard analysis were used to perform time-to-event analysis. A p value less than 0.05 was considered statistically significant. All the analyses were conducted using R (version 4.1.0; R Development Core Team, Vienna, Austria).

## Results

Patient characteristics

The baseline characteristics of the 400 patients are summarized in Table 1. Notably, the included patients had a mean age of 70.7 years, with 60.0% being male. All patients were at least 28 years old. Exacerbating factors at the time of hospitalization for HF were divided into four major categories: ischemic cardiomyopathy (ICM), cardiomyopathy without ICM (e.g., hypertensive cardiomyopathy (HHD), tachycardia-induced cardiomyopathy (TIC), cardiac sarcoidosis (CS), dilated cardiomyopathy (DCM)), valve disease, others (e.g., hyperthyroidism, pulmonary hypertension without left heart disease). Those who were divided into ICM include patients with past history of ICM or diagnosed after examination following hospitalization. There were no significant differences in HF with ICM and valve disease but with significant differences in HF with cardiomyopathy without ICM. No significant differences in physical examination findings and medical history were observed between the groups. Regarding medications upon discharge, tolvaptan tended to be used more often in patients with pure RBBB than in those with no BBB and LBBB. No significant differences in the use of other oral medications were observed.

**Table 1:** Characteristics of no BBB, pure RBBB, and LBBB at the time of discharge (n=400) Values are expressed as n (%) or mean ± standard deviation, median (interquartile range). Abbreviations: BMI = body mass index; BW = body wight; HF = heart failure; ICM = ischemic cardiomyopathy; BP = blood pressure; NYHA = New York Heart Association; HD = hemodialysis; PD = peritoneal dialysis; ACE-I = angiotensin-converting enzyme inhibitor; ARB = angiotensin 2 receptor blocker; MRA = mineralocorticoid receptor antagonist; CCB = calcium channel blocker; DPP-4 I = dipeptidyl peptidase-4 inhibitor; SGLT-2 I = sodium glucose cotransporter-2 inhibitor; DOAC = direct oral anticoagulants

Variables	Number of data	No BBB (n=307)	Pure RBBB (n=37)	LBBB (n=56)	p value
Age	400	70.6 ± 13.8	72.4 ± 12.4	70.6 ± 13.1	0.66
Male	400	181 (59.0%)	23 (62.2%)	36 (64.3%)	0.73
BMI (kg/m^2^)	379	21.8 ± 4.4	21.0 ± 3.4	22.9 ± 3.9	0.36
BW (kg)	388	56.7 ± 15.2	53.6 ± 12.5	58.9 ± 12.9	0.87
Smoker/ex-smoker	393	177 (58.8%)	22 (61.1%)	38 (67.9%)	0.44
HF with ICM	400	102 (33.2%)	14 (33.2%)	24 (42.9%)	0.34
HF with cardiomyopathy without ICM	400	147 (47.9%)	8 (21.6%)	22 (39.3%)	0.006
HF with valve disease	400	41 (15.3%)	10 (27.0%)	9 (16.1%)	0.09
Systolic BP (mmHg)	400	111.8 ± 16.8	111.5 ± 16.2	110.5 ± 17.5	0.79
Diastolic BP (mmHg)	400	60.5 ± 10.6	58.0 ± 12.0	59.1 ± 9.4	0.57
NYHA class III-IV	397	233 (77.6%)	26 (70.3%)	47 (83.9%)	0.29
Medical history					
Hypertension	400	166 (54.1%)	20 (54.1%)	33 (58.9%)	0.8
Diabetes mellites	400	102 (33.2%)	19 (51.4%)	23 (41.1%)	0.067
Dyslipidemia	400	59 (19.2%)	8 (21.6%)	10 (17.9%)	0.9
Heart failure	400	46 (15.0%)	8 (21.6%)	11 (19.6%)	0.45
Ischemic heart disease	400	57 (18.6%)	6 (16.2%)	12 (21.4%)	0.781
Cerebral infarction	400	29 (9.4%)	3 (8.1%)	6 (10.7%)	0.92
Chronic kidney disease	400	51 (16.6%)	8 (21.6%)	9 (16.1%)	0.73
HD/PD	400	15 (4.9%)	3 (8.1%)	2 (3.6%)	0.61
Baseline medication					
ACE-I/ARB	400	208 (67.8%)	25 (67.6%)	41 (73.2%)	0.72
MRA	400	192 (62.5%)	23 (62.2%)	39 (69.6%)	0.59
β-blocker	400	272 (88.6%)	28 (75.8%)	47 (83.9%)	0.073
CCB	400	88 (28.7%)	10 (27.0%)	14 (25.0%)	0.85
Loop diuretic	400	207 (67.4%)	26 (70.3%)	39 (69.6%)	0.9
Tolvaptan	400	61 (19.9%)	14 (38.9%)	10 (17.9%)	0.034
Statin	400	92 (30.0%)	13 (35.1%)	20 (35.7%)	0.6
DPP-4 I	400	43 (14.0%)	7 (12.5%)	9 (16.1%)	0.7
SGLT-2 I	400	3 (1.0%)	0 (0%)	0 (0%)	0.63
Pimobendan	400	14 (4.6%)	3 (8.1%)	2 (3.6%)	0.57
DOAC or Warfarin	400	151 (49.2%)	17 (45.9%)	22 (39.3%)	0.39

Laboratory findings and physiological examination

Baseline laboratory and physiological examination findings are detailed in Table 2. Accordingly, although no significant differences in transaminase, creatinine, and brain natriuretic peptide levels were noted, the pure RBBB group had significantly lower levels of baseline hemoglobin. With regard to ECG findings, fewer patients in the no BBB group than in the other two groups had a sinus rhythm and normal QTc duration. In terms of echocardiography findings, the pure RBBB group had fewer patients having HF with reduced ejection fraction (HFrEF) and more patients with the EF mean value than the other groups. Conversely, the LBBB group tended to have more patients with left ventricular enlargement than did the no BBB and pure RBBB groups.

**Table 2:** Examination findings of no BBB, pure RBBB, and LBBB at the time of discharge (n=400) Values are expressed as n (%) or mean ± standard deviation, median (interquartile range). Abbreviations: AST = aspartate aminotransferase; ALT = alanine aminotransferase; γ-GTP = γ-glutamyl transpeptidase; LDH = lactate dehydrogenase; BUN = blood urea nitrogen; eGFR = estimated glomerular filtration rate; BNP = brain natriuretic peptide; LVDd = left ventricular end-diastolic diameter; LVDs = left ventricular end-systolic diameter; HFrEF = heart failure with reduced ejection fraction

Variables	Number of data	No BBB (n=307)	Pure RBBB (n=37)	LBBB (n=56)	p value
Laboratory data					
Sodium	400	138.4 ± 3.7	138.8 ± 3.4	138.4 ± 3.5	0.79
Potassium	400	4.3 ± 0.5	4.3 ± 0.5	4.3 ± 0.5	0.73
Chloride	400	103.6 ± 4.2	104.2 ± 4.7	103.9 ± 4.0	0.59
AST	399	27.6 ± 16.7	24.6 ± 11.3	25.1 ± 15.4	0.26
ALT	399	27.6 ± 27.7	19.2 ± 13.0	21.9 ± 19.0	0.092
γ-GT	347	61.9 ± 75.8	75.0 ± 91.2	43.1 ± 28.4	0.46
LDH	398	226.1 ± 65.7	255.1 ± 158.3	213.7 ± 56.6	0.28
BUN	400	26.3 ± 12.7	28.6 ± 19.6	26.7 ± 12.6	0.81
Creatinine	400	1.55 ± 1.81	1.40 ± 1.15	1.65 ± 1.95	0.55
eGFR	400	48.7 ± 22.0	49.7 ± 24.5	46.1 ± 20.0	0.68
Hemoglobin	400	12.6 ± 2.6	11.3 ± 2.0	12.8 ± 2.4	0.018
BNP	391	369.1 ± 450.1	358.9 ± 273.9	452.3 ± 519.1	0.2
Electrocardiogram					
Sinus rhythm	400	178 (58.0%)	26 (72.2%)	44 (78.6%)	0.0008
Heart rate (bpm)	400	101.7 ± 29.4	90.1 ± 28.3	101.9 ± 23.1	0.062
QRS duration (ms)	381	88.2 ± 9.8	132.7 ± 19.8	124.6 ± 20.3	<0.001
QTc duration (ms)	380	431.2 ± 38.6	463.3 ± 40.6	464.1 ± 47.7	<0.001
Ultrasonic cardiogram					
LVDd (mm)	392	54.0 ± 8.7	52.2 ± 10.6	61.6 ± 11.3	<0.001
LVDs (mm)	392	41.0 ± 11.0	36.7 ± 10.5	50.2 ± 11.7	<0.001
Ejection fraction (%)	394	47.2 ± 17.6	54.4 ± 17.3	37.3 ± 13.5	<0.001
HFrEF	394	127 (41.9%)	9 (24.3%)	34 (63.0%)	<0.001

Outcomes

A comparison between the no BBB, pure BBB, and LBBB groups showed significant differences in MACE and rehospitalization due to HF (Table 3). Meanwhile, to evaluate the prognosis of pure RBBB, a comparison between the no BBB and pure RBBB groups showed that pure RBBB was independently associated with MACE (odds ratio: 2.83 (95% confidence interval: 1.31-6.12); p = 0.008; Table 4) and HF rehospitalization (odds ratio: 3.01 (95% confidence interval): (1.34-6.77); p = 0.008; Table 5) even after adjusting for classical HF risk factors in the multivariable logistic model. Table 6 shows multivariable-adjusted hazard ratios of no BBB and pure RBBB for MACE and HF rehospitalization adjusting for classical HF risk factors. Patients with RBBB had increased risk of MACE (hazard ratio: 2.02 (95% confidence interval: 1.18-3.45); p = 0.01) and HF rehospitalization (hazard ratio: 2.40 (95% confidence interval: 1.26-4.58); p = 0.008).

**Table 3:** Outcomes of no BBB, pure RBBB, and LBBB at the time of discharge (n=400) Values are expressed as n (%) or mean ± standard deviation, median (interquartile range). Abbreviations: BBB = bundle branch block; RBBB = right bundle branch block; LBBB = left bundle branch block; MACE = major adverse cardiovascular events; HF = heart failure

Variables	Number of data	NoBBB (n=307)	Pure RBBB (n=37)	LBBB (n=56)	p value
MACE	400	80 (26.1%)	19 (51.4%)	21 (37.5%)	0.003
All causes of death	400	28 (9.1%)	7 (18.9%)	7 (12.5%)	0.144
HF rehospitalization	400	51 (16.6%)	13 (35.1%)	16 (28.6%)	0.007
Myocardial infarction	400	5 (1.6%)	1 (2.7%)	1 (1.8%)	0.65
Cerebral apoplexy	400	5 (1.6%)	1 (2.7%)	0 (0%)	0.57

**Table 4:** Multivariate analysis model evaluating the impact of no BBB and pure RBBB on MACE adjusting for classical risk factors for HF (n=344, 99 MACE) Abbreviations: BBB = bundle branch block; RBBB = right bundle branch block; MACE = major adverse cardiovascular events; HF = heart failure; BMI = body mass index; eGFR = estimated glomerular filtration rate Classical HF risk factors: body mass index; hemoglobin level; estimated glomerular filtration rate level; albumin level

Variable	Unit of comparison	OR	95% CI	p value
Pure RBBB	Presence vs. absence	2.83	1.31-6.12	0.008
BMI	Per 1 m^2^/kg	0.97	0.92-1.02	0.29
Hemoglobin	Per 1 g/dL	0.80	0.70-0.93	0.002
eGFR	Per 1 mL/min/1.73 m^2^	0.99	0.70-1.01	0.37
Albumin	Per 1 g/dL	1.01	0.57-1.78	0.98

**Table 5:** Multivariate analysis model evaluating the impact of no BBB and pure RBBB on HF rehospitalization adjusting for classical risk factors for HF (n=344, 64 HF rehospitalization) Abbreviations: BBB = bundle branch block; RBBB = right bundle branch block; HF = heart failure; BMI = body mass index; eGFR = estimated glomerular filtration rate Classical HF risk factors: body mass index; hemoglobin level; estimated glomerular filtration rate level; albumin level

Variable	Unit of comparison	OR	95% CI	p value
Pure RBBB	Presence vs. absence	3.01	1.34-6.77	0.008
BMI	Per 1 m^2^/kg	0.98	0.92-1.04	0.46
Hemoglobin	Per 1 g/dL	0.90	0.77-1.05	0.17
eGFR	Per 1 mL/min/1.73 m^2^	0.99	0.98-1.00	0.13
Albumin	Per 1 g/dL	1.50	0.78-2.88	0.23

**Table 6:** Hazard ratios of no BBB and pure RBBB for MACE and HF rehospitalization at one year follow-up adjusting for classical risk factors Abbreviations: BBB = bundle branch block; RBBB = right bundle branch block; MACE = major adverse cardiovascular events; HF = heart failure Classical HF risk factors: body mass index; hemoglobin level; estimated glomerular filtration rate level; albumin level

Variable	HR	95% CI	p value
MACE	2.02	1.18-3.45	0.01
HF rehospitalization	2.40	1.26-4.58	0.008

**Figure 2:**
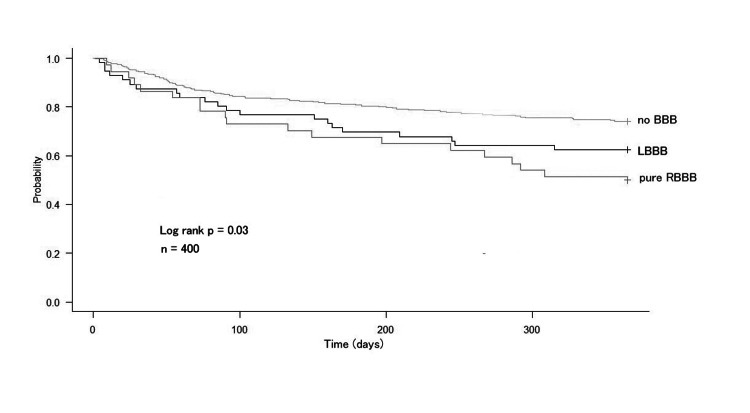
Survival to MACE compared by bundle branch block Abbreviations: BBB = bundle branch block; LBBB = left bundle branch block; RBBB = right bundle branch block; MACE = major adverse cardiovascular events

**Figure 3:**
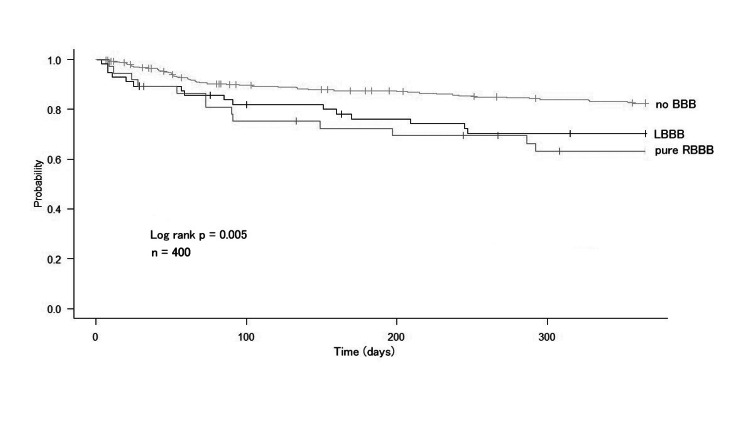
Survival to heart failure rehospitalization compared by bundle branch block Abbreviations: BBB = bundle branch block; LBBB = left bundle branch block; RBBB = right bundle branch block

Figure 2 shows the Kaplan-Meier curves for MACE in the no BBB, pure RBBB, and LBBB groups. The unadjusted risk for MACE was the highest in the pure RBBB group, followed by the LBBB group. At one year, 51% (n = 19), 26% (n = 80), and 38% (n = 21) of patients with pure RBBB, no BBB, and LBBB experienced MACE, respectively. Figure 3 shows the Kaplan-Meier curves for HF rehospitalization in the no BBB, pure RBBB, and LBBB groups. At one year, 35% (n = 13), 17% (n = 51), and 29% (n = 16) of patients with pure RBBB, no BBB, and LBBB were hospitalized for HF.

## Discussion

The current study found that the presence of pure RBBB was associated with a higher frequency MACE and HF rehospitalization. A wide QRS duration has been considered a risk for worsening prognosis, such as mortality or HF rehospitalization in patients with HF [3,16,17]. Most previous studies have shown that the presence of RBBB requiring no treatment was associated with improved prognosis [5,18]. On the contrary, recent studies have found that incidentally discovered RBBB may increase the risk of mortality in patients with no CVD [7,18]. Our study supports the report that found a worse prognosis among those with a wide QRS duration and provides new findings showing that the presence of RBBB increases the risk of not only MACE but also rehospitalization due to HF. Our findings showed that the prevalence of RBBB was 12.9%, which is higher than that in the general population [7,18]. Heart muscle fibrosis caused by hypertension, ischemic heart disease, or pulmonary hypertension, such as pulmonary thromboembolism, can facilitate the development of RBBB [5,6]. These background diseases have been associated with the onset of HF. Therefore, patients with HF may be at increased risk for RBBB occurrence compared to healthy individuals [19].

The mechanism by which RBBB is associated with a worse prognosis is not clear at this time. Barsheshet et al. pointed out that, in patients with systolic HF, the presence of RBBB caused a delay in left ventricular contractile activity, as did LBBB [20]. They stated that this may reflect extensive myocardial damage, including not only right ventricular but also left ventricular, in patients with RBBB. Inciardi et al. also stated that right ventricular dysfunction and pulmonary hypertension associated with increased left ventricular filling pressures put patients at risk for heart failure, regardless of left ventricular contractility or diastolic capacity [21]. Barsheshet et al. found that patients with left systolic HF and RBBB had pulmonary hypertension with worse right ventricular function [8]. Left and right ventricular functions have been closely related, with left HF progression leading to biventricular HF comprising right HF, which consequently increases biventricular HF with right heart load, suggesting that the duration of HF may impact the development of RBBB and prognosis [8,22]. The right bundle branch is thinner than the left bundle branch and is known to be more prone to ischemia or load-induced tearing, making it more susceptible to pulmonary hypertension due to left heart failure at an earlier stage [23], which may reflect that the HF status is so poor that RBBB is chronically placed in a state of pulmonary hypertension. However, we failed to assess these associations given the limited or missing data on HF duration or Swan-Ganz catheter pressure.

There were significant differences between the groups with left ventricular enlargement and left ventricular systolic function, and cause of HF, there was a trend toward more valvular disease in the pure RBBB group and more ICM in the LBBB group. This was not the authors' intention; left ventricular enlargement and low left ventricular contractility were more common in LBBB and may be caused by ICM, and the possibility that the cause of HF had an impact on prognosis cannot be denied. On the other hand, there is no conclusion that has been reached whether HF with reduced or preserved left ventricular contractility has a poorer prognosis [24,25]. Patients with pure RBBB had few HFrEF and tended to have low average heart rates compared with other groups. A recent study shows that, in patients with HFpEF, the usage of β-blocker is the risk of HF rehospitalization, because of bradycardia or chronotropic incompetence [26]. The usage of β-blocker in patients with pure RBBB might be one of the reasons for the poor prognosis of RBBB in our study. The reason for the significant differences in cardiomyopathy without ICM, which is an exacerbating factor for HF, was unclear. More details about cardiomyopathies were reviewed, but due to the small number of cases, an obvious cause could not be identified.

Previous studies show that QRS duration itself may have been strongly involved in the prognosis [7,27]. LBBB and a wide QRS duration occur as adaptations to CRT because of the risk of all-cause death or HF rehospitalization, whereas CRT has been found to promote no improvements in the prognosis of patients with RBBB [28]. This could be attributed to the fact that CRT improves the synchrony of the left ventricle but may worsen the synchrony of the right ventricle [28]. Bundle branch block is defined as prolonged QRS duration, but the pathophysiology differs between RBBB and LBBB. Additionally, it has been reported that electrical dyssynchrony, which is related to QRS time, does not correlate with mechanical dyssynchrony, which is related to visual images such as echocardiography [29]. It is important to understand the pathophysiological differences between RBBB and LBBB to treat HF accordingly, not lumping them together as QRS duration prolongation.

While patients with pure RBBB tend to show low hemoglobin levels, there were no significant differences from the other groups in indices related to malnutrition, renal dysfunction, or background disease that could cause anemia. Therefore, the authors could not explain why there was a significant difference in hemoglobin levels; it is possible that the higher prevalence of anemia in patients with pure RBBB reflected the severity of HF [15]. In addition, patients with pure RBBB tend to be used tolvaptan suggesting that stronger fluid control with diuretics may have been more necessary. It had been reported that tolvaptan use is a severity index for HF rehospitalization and using tolvaptan in the current study may represent a severity of HF [30].

Notably, our Kaplan-Meier analysis, which evaluated MACE and HF rehospitalization at one year, revealed that the LBBB group had a worse prognosis than the no BBB group but a similar prognosis to the pure RBBB. Interestingly, our findings showed that the pure RBBB group had a higher risk of having a poorer prognosis, similar to the LBBB group, than did the no BBB group. This suggests that the presence of RBBB may be an indicator of poor prognosis in patients with HF and may be beneficial in identifying patients requiring careful observation.

Limitations

This was a small group and single-center retrospective study with missing baseline characteristics. The occurrence of RBBB was also collected retrospectively from medical records. Therefore, some incidences during hospitalization might have been overlooked. Moreover, a selection bias may have occurred in this study given that in-hospital death, cases with less than one year of observation, and patients with LBBB-introduced CRT were excluded. There were significant differences between BBBs in hemoglobin level, tolvaptan use, and exacerbating factors for HF, which may result in confounding bias. Lack of the number of heart failure exacerbations during a lifetime may have influenced prognosis assessment. These missing data may have affected our results, particularly with respect to RBBB outcomes. Our models did not adjust for exacerbation times or the duration of HF. Therefore, the impact of exacerbation times or HF duration on the outcomes could not be evaluated. Further studies are needed to address these limitations. We did not evaluate right heart catheterization considering that RBBB is caused by increased right heart load. We cannot confirm whether RBBB occurred with or without right heart loading. Although the prevalence RBBB has been considered to increase with age, the influence of age on RBBB onset has not been evaluated.

## Conclusions

The current study has described the prognosis of patients with HF classified by ECG findings. Patients with pure RBBB had a higher rate of MACE and HF rehospitalization compared with no BBB after discharge at one year. According to Kaplan-Meier survival curves, patients with pure RBBB had a poor prognosis compared with no BBB, which was equivalent to LBBB. Patients with pure RBBB may have a poor prognosis as well as LBBB, and these patients require careful medication management and understanding the differences between RBBB and LBBB pathologies in outpatient settings to prevent rehospitalization.
